# Changes in Neural Activity Underlying Working Memory after Computerized Cognitive Training in Older Adults

**DOI:** 10.3389/fnagi.2016.00255

**Published:** 2016-11-08

**Authors:** Erich S. Tusch, Brittany R. Alperin, Eliza Ryan, Phillip J. Holcomb, Abdul H. Mohammed, Kirk R. Daffner

**Affiliations:** ^1^Laboratory of Healthy Cognitive Aging, Center for Brain/Mind Medicine, Division of Cognitive and Behavioral Neurology, Department of Neurology, Brigham and Women’s Hospital, Harvard Medical School, BostonMA, USA; ^2^Department of Psychology, Oregon Health and Science University, PortlandOR, USA; ^3^Department of Psychology, Tufts University, MedfordMA, USA; ^4^Department of Psychology, Linnaeus UniversityVäxjö, Sweden; ^5^Center for Alzheimer Research, Department of Neurobiology, Care Sciences and Society, Karolinska InstitutetHuddinge, Sweden

**Keywords:** computerized cognitive training, working memory, ERPs (Event-Related Potentials), cognitive aging, P3a, P3b, n-back task, P3

## Abstract

Computerized cognitive training (CCT) may counter the impact of aging on cognition, but both the efficacy and neurocognitive mechanisms underlying CCT remain controversial. In this study, 35 older individuals were randomly assigned to Cogmed adaptive working memory (WM) CCT or an active control CCT, featuring five weeks of five ∼40 min sessions per week. Before and after the 5-week intervention, event-related potentials were measured while subjects completed a visual n-back task with three levels of demand (0-back, 1-back, 2-back). The anterior P3a served as an index of directing attention and the posterior P3b as an index of categorization/WM updating. We hypothesized that adaptive CCT would be associated with decreased P3 amplitude at low WM demand and increased P3 amplitude at high WM demand. The adaptive CCT group exhibited a training-related increase in the amplitude of the anterior P3a and posterior P3b in response to target stimuli across n-back tasks, while subjects in the active control CCT group demonstrated a post-training decrease in the anterior P3a. Performance did not differ between groups or sessions. Larger overall P3 amplitudes were strongly associated with better task performance. Increased post-CCT P3 amplitude correlated with improved task performance; this relationship was especially robust at high task load. Our findings suggest that adaptive WM training was associated with increased orienting of attention, as indexed by the P3a, and the enhancement of categorization/WM updating processes, as indexed by the P3b. Increased P3 amplitude was linked to improved performance; however. there was no direct association between adaptive training and improved performance.

## Introduction

### Computerized Cognitive Training (CCT)

Promoting healthy cognitive aging is a major public health goal. According to a recent UN report (United Nations, 2013), individuals over the age of 60 are the fastest growing age group on earth. Studies have suggested more than half of adults over the age of 65 have concerns about their memory (Bassett and Folstein, 1993; Ponds et al., 1997; Commissaris et al., 1998). Losing one’s mental faculties and independence are among the most feared aspects of getting older. Moreover, caring for older individuals who can no longer manage independently has become a leading public health challenge. In response to these concerns, there is growing interest in developing strategies or interventions that augment intellectual health and for understanding the neural mechanisms that underlie improvement in performance on cognitive tasks. These efforts seem particularly relevant, given the recent preliminary report suggesting that processing speed training in older adults may reduce the risk of developing dementia 10 years later (Edwards et al., 2016).

The sale of computerized cognitive training (CCT) programs is approaching $1 billion per year (The-Economist, 2013), These programs are often marketed toward older adults as reflecting sound scientific evidence, when in fact there has been relatively little systematic study (Kueider et al., 2012; Green et al., 2014; Lampit et al., 2014). Recently, more doubt has been cast on consumer-focused CCT, including one popular firm having been formally reprimanded for false claims (Federal Trade Comission, 2016). Nevertheless, interest in “brain training” remains high. A recent consensus statement on the “brain training industry” organized by Stanford University and Max Planck Institute (Max Planck Institute, 2014) emphasized the need for much more research by investigators with no financial interest in the products, who will conduct rigorously designed studies that include a control group treated exactly the same as the trained group, except for the specific training. A varied body of research (Shipstead et al., 2012; Zinke et al., 2012; Melby-Lervag and Hulme, 2013; Spencer-Smith and Klingberg, 2015) has been conducted to investigate the effectiveness of CCT. Of particular interest is the degree to which CCT affects performance on un-trained tasks (i.e., transfer effects). A critical aspect of this research is to compare an adaptive CCT condition, wherein task difficulty is continuously modulated based on performance, against an active control CCT condition. In the current study, the control CCT group performed the same tasks as the adaptive CCT group, but task difficulty was constant over the entire training period (see Methods for details). The use of adaptive CCT is important for ensuring task engagement and the validity of group differences (Karbach and Kray, 2009; Shipstead et al., 2012).

Recent studies have presented mixed results on the effects of CCT in older adults (Jaeggi et al., 2008; Brehmer et al., 2012; Zinke et al., 2012). For example, Brehmer et al. (2011) included an adaptive and control CCT group using the same interventions as the current study, and showed that cortical decreases in fMRI activation, paired with subcortical increases in activity, were associated with the largest training-related performance gains on an un-trained task. The augmented subcortical activity was interpreted as reflecting operations of WM becoming more automated or proceduralized. Belleville et al. (2014) tested performance on trained tasks after simple and complex training, and measured fMRI activity in areas associated with WM. They found decreased neural activity after simple training on repetitive tasks, but increased activity after training on more complex tasks involving flexible control over attentional resources. Vermeij et al. (2016), using functional near infrared spectroscopy (fNIRS) to assess hemodynamic responses in left and right prefrontal cortex, found decreased activity during high load untrained tasks. Together with unchanged performance, this pattern is often interpreted as reflecting improved, more efficient processing (Heinzel et al., 2014; Vermeij et al., 2016).

In contrast to the preceding research that employed fMRI and fNIRS to investigate WM processes, the current study measured event-related potentials (ERPs). WM and attentional processes were indexed by the P3 component (Wickens et al., 1983; Polich, 1996) in response to target stimuli during an n-back task. The P3 reflects the activity of two sub-components, the P3a and P3b (Snyder and Hillyard, 1976; Polich, 2007). The P3a is an anteriorly distributed component usually peaking between 300 and 500 ms that has been interpreted as an index of executive control processes such as evaluating events or tasks to determine whether they merit additional processing or action, or as a marker of the orienting of attention to a stimulus or task (Daffner et al., 1998, 2003; Friedman et al., 2001; Barcelo et al., 2002; Dien et al., 2004; Barcelo et al., 2006; Alperin et al., 2014). The P3b is a central-posteriorly distributed component usually peaking between 400 and 600 ms that has been interpreted as an index of the categorization process or working memory (WM) updating after categorization has taken place (Donchin, 1981; Donchin et al., 1986; Knight and Scabini, 1998; Kok, 2001; Verleger et al., 2005; Daffner et al., 2011a).

### Objectives and Hypotheses

The current study had two major objectives. The first goal was to determine whether CCT in older adults was associated with improvement of behavioral performance on an untrained task (i.e., transfer effects). The second goal was to identify the neural mechanisms underlying change in those adults whose behavioral performance improved after CCT. Recent studies have investigated the effects of training on prospective memory (Rose et al., 2015) and speed of processing (O’Brien et al., 2013), but surprisingly little is known about changes in neural processes that mediate improvement in WM task performance in older adults, which is an important prerequisite for designing future interventions.

**FIGURE 1:**
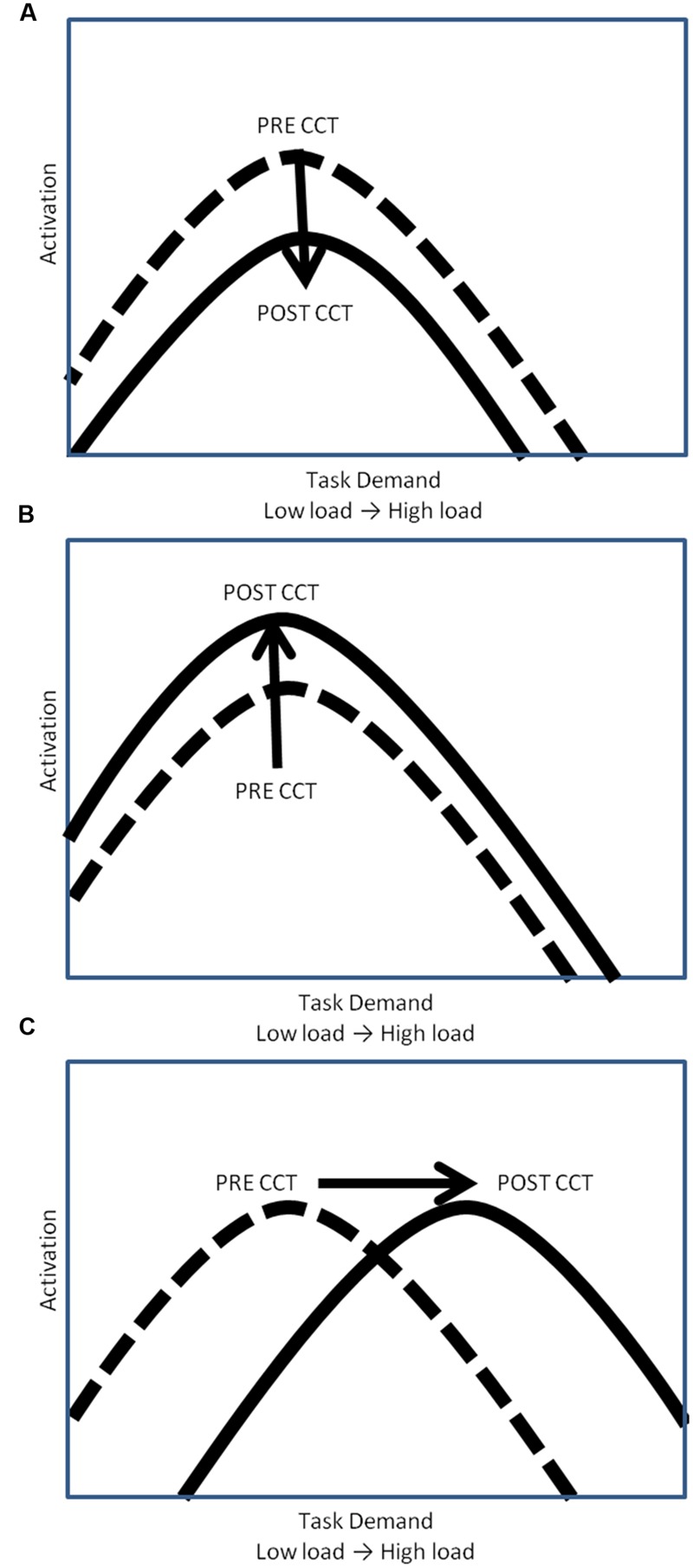
**Hypothetical task-demand/resource utilization curves. (A)** Downward shift of hypothetical task demand-activation curve. **(B)** Upward shift of hypothetical task demand-activation curve. **(C)** Rightward shift of hypothetical task demand-activation curve.

The relationship between WM task demands and resource utilization (i.e., neural activation) has been conceptualized by the compensation-related utilization of neural circuits hypothesis (CRUNCH) (Reuter-Lorenz and Cappell, 2008; Schneider-Garces et al., 2010) in terms of an inverted U-shaped curve. According to CRUNCH, activation increases with task demands until the subjects’ maximum processing capacity has been reached (the ‘crunch’ point), at which time activation decreases as load continues to increase (see **Figure 1**) (Reuter-Lorenz and Cappell, 2008; Schneider-Garces et al., 2010). CCT may modulate this task-demand/resource utilization curve in one of three ways. One possibility is that CCT would reduce overall activation, thereby shifting the task-demand/resource utilization curve downward (**Figure 1A**). Decreased activation coupled with either unchanged or improved performance can be interpreted as reflecting more efficient processing (Doyon and Benali, 2005; Brehmer et al., 2011; Belleville et al., 2014; Heinzel et al., 2014; Vermeij et al., 2016).

Computerized cognitive training may also lead to an increase in activation, thereby shifting the task-demand/resource utilization curve upward (**Figure 1B**). In this scenario, CCT training would be associated with greater appropriation of processing resources at all levels of task load. Increased recruitment of resources to match task demands should be associated with concomitant improvement in behavioral performance. Increased activation in the absence of behavioral improvements could be understood as inefficient utilization of resources. A final possibility is that CCT would shift the task-demand/resource utilization curve to the right (**Figure 1C**). In this scenario, individuals would execute low load tasks utilizing fewer resources (greater efficiency) but be able to appropriate more resources (greater neural activity) in response to high task load conditions. Based on past work, we expected neural activation during the untrained task, as indexed by P3 amplitude, to increase after CCT on high load tasks, but decrease on low load tasks, represented by a shift to the right on the task-demand/resource utilization curve (Daffner et al., 2011a). Behaviorally, we expected transfer effects in the adaptive CCT group, leading to improved n-back performance after CCT. We anticipated no changes in either activation or performance after active control CCT.

## Materials and Methods

### Participants

Subjects were recruited through community announcements in the Boston metropolitan area. The study was approved by the Partners Human Research Committee (protocol number 2013P002266). All subjects completed written informed consent. Subjects also completed a detailed screening evaluation that included a structured interview to obtain a medical, neurological, and psychiatric history; a formal neurological examination and test of visual acuity via Snellen Wall chart; and the completion of a neuropsychological test battery and questionnaires surveying mood and daily living activities.

To be included in this study, participants had to be English-speaking, have ≥12 years of education, have a Mini Mental State Exam (MMSE) (Folstein et al., 1975) score ≥26, and an estimated intelligence quotient (IQ) on the American National Adult Reading Test (AMNART) (Ryan and Paolo, 1992) ≥100. Subjects were excluded if they had a history of CNS diseases or major ongoing psychiatric disorders based on DSM-IV criteria (American Psychiatric Association, 1994), focal abnormalities on neurological examination consistent with a CNS lesion, or a history of clinically significant medical diseases. Clinical history and baseline performance on neuropsychological tests allowed us to exclude subjects with evidence of dementia (American Psychiatric Association, 1994) or mild cognitive impairment (MCI; Petersen et al., 1999). Subjects were randomly assigned to either adaptive or active control CCT groups, and completed the same set of tasks, tests, and questionnaires.

### Experimental Procedure

**FIGURE 2:**
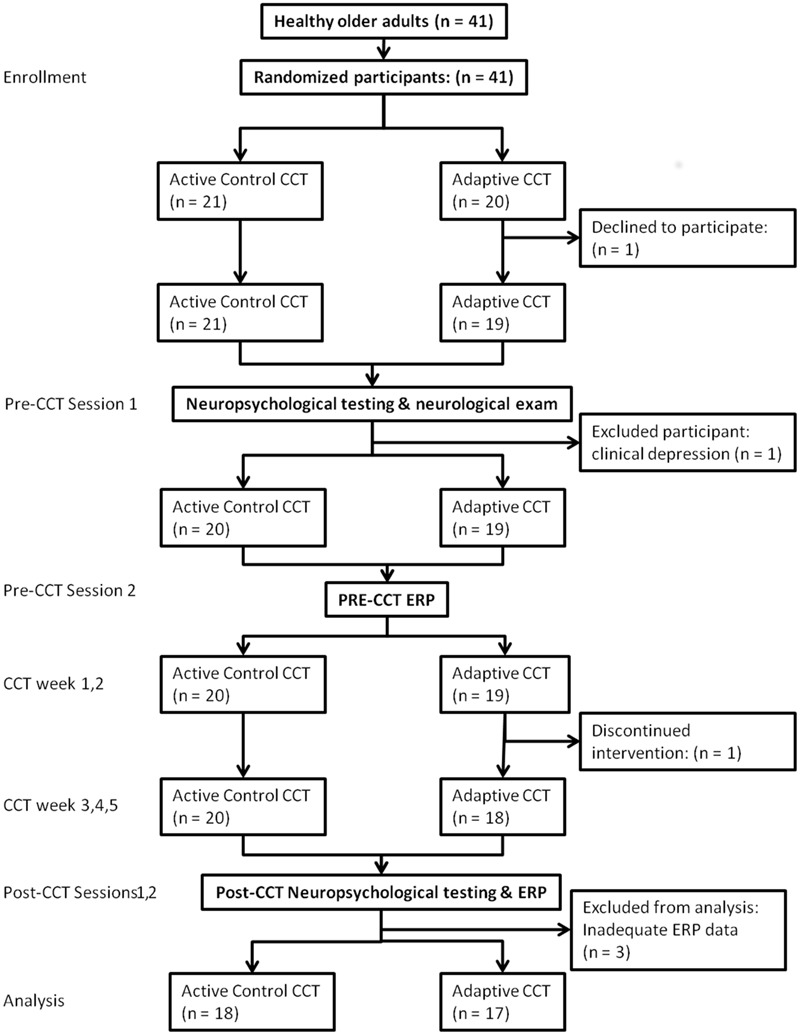
**Subject flow chart**.

Subject flow is shown in **Figure 2** according to the CONSORT reporting instructions (Schulz et al., 2010). Subjects completed two pre-CCT and two post-CCT visits to the laboratory. During the first pre-CCT visit, subjects completed neuropsychological testing and neurological examination. During the second pre-CCT visit, subjects performed the experimental n-back task while ERPs were collected. Neuropsychological testing and the experimental task were repeated during two post-CCT visits, which were scheduled as close as possible to the end of each subjects’ CCT period (CCT to post-CCT testing days elapsed: *mean* = 3.86).

Subjects completed the following neuropsychological measures during their pre-CCT lab visit: (1) MMSE (Folstein et al., 1975), a brief measure of overall cognitive status; (2) AMNART (Ryan and Paolo, 1992), providing an estimate of IQ; (3) WMS-III Logical Memory subtest (Wechsler, 1997), which indexes episodic memory; (4) Boston Naming Test (Lansing et al., 1999) which is a measure of language function, specifically word retrieval; (5) Trail-making test parts A and B (Reitan and Wolfson, 1985), which measure planning/sequencing, set shifting, and inhibition; (6) WAIS-IV Digit-Symbol Coding (Wechsler, 2008), which assesses monitoring, inhibition, and manipulation; (7) Controlled Oral Word Association Test (COWAT) (Ivnik et al., 1996), which indexes initiation, self-generation, and monitoring. All test scores except MMSE and AMNART were scaled against age-matched percentile norms in order to eliminate any performance differences due to the age range of subjects (Daselaar and Cabeza, 2005; Riis et al., 2008; Daffner et al., 2011a).

**FIGURE 3:**
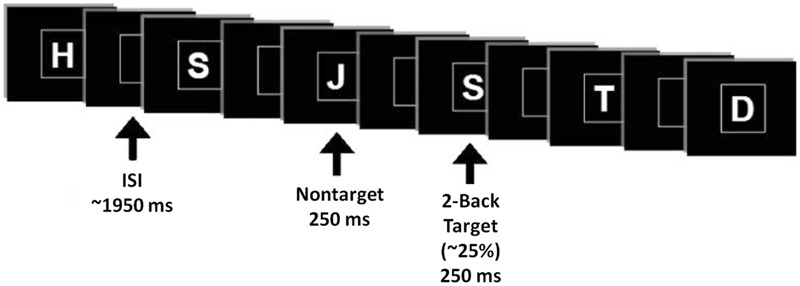
**N-Back task illustration**.

Event-related potentials were collected while participants performed a verbal n-back paradigm in the visual modality with three levels of difficulty (0-back, 1-back, and 2-back). Stimuli consisted of letters of the alphabet, white on a black background, presented within a square at the center of a high resolution computer monitor for 250 ms, in pseudorandom order (2-back task demonstrated in **Figure 3**). Under each n-back condition, subjects were shown a series of 300 letters, divided into three blocks. For each level of n-back, 75% of trials were non-matches and 25% of trials were matches. Subjects were instructed to respond as quickly as possible without sacrificing accuracy, via mouse click, only to match letters. The inter-stimulus interval varied randomly between 1850 and 2050 ms (mean ∼1950 ms), and the square encompassing stimuli remained on the screen at all times. The hand used for mouse click and the order of n-back tasks were counterbalanced across subjects.

### Intervention

Cogmed, a commercially available computerized WM CCT program (Cogmed QM, Pearson Education, Inc.), was utilized in this study. Subjects completed either adaptive or active control WM CCT for 5 weeks between their first and second testing sessions. Time commitments were equivalent for both CCT types; subjects were instructed to train five days per week for five weeks. Individual training sessions lasted approximately 40 min and consisted of eight tasks automatically selected from a set of twelve verbal and visuospatial WM tasks. Subjects were instructed to perform all tasks within one block of time with minimal breaks between tasks. Subject progress was monitored by study personnel and subjects were contacted at least once per week in order to address questions or concerns and to provide encouragement. No training strategies were offered by study personnel.

Most Cogmed tasks emphasize the maintenance aspects of verbal and spatial WM. A few emphasize both maintenance and manipulation aspects of WM. No Cogmed tasks emphasize rapid, continuous updating of the contents of WM, as is required by the experimental n-back task under the 1-back and 2-back loads. Under adaptive CCT, task difficulty was revised on a trial-by-trial basis with the goal of establishing 60% accuracy, thereby creating a consistently challenging level of subjective difficulty for each individual subject. This is important not only to foster engagement, but to drive any possible training effects (Karbach and Kray, 2009; Shipstead et al., 2012; Vermeij et al., 2016). Task difficulty was modulated by increasing or decreasing the WM load for each trial, e.g., the number of letters to keep in mind. Under active control CCT, task difficulty remained at a constant, relatively low load across all training days.

### ERP Recordings

During the pre- and post-training testing sessions, an ActiveTwo electrode cap (Behavioral Brain Sciences Center, Birmingham, UK) was used to hold to the scalp a full array of 128 Ag-AgCl BioSemi (Amsterdam, Netherlands) “active” electrodes whose locations were based on a pre-configured montage. Electrodes were arranged in equidistant concentric circles from 10 to 20 system position Cz. In addition to the 128 electrodes on the scalp, six mini bio-potential electrodes were placed over the left and right mastoid, beneath each eye, and next to the outer canthi of the eyes to check for eye blinks and vertical and horizontal eye movements. EEG activity was digitized at a sampling rate of 512 Hz and filtered off-line with a band-pass filter of 0.016-100 Hz.

### Data Analysis

#### Behavioral Analyses

Performance on the 0-back, 1-back, and 2-back task was analyzed via a method adapted from Vermeij et al. (2016). While Vermeij et al. (2016) featured an additional n-back task (3-back) and included adults with MCI, the overarching structure of the study was mostly consistent with the present study; both featured Cogmed adaptive and active control training groups and tested older adults. The nonparametric discrimination index (i.e., sensitivity) A′ was calculated. A′ is a behavioral performance variable derived from signal detection theory (Grier, 1971; Hannay, 1988) and ranges from 0.5 (chance level) to 1 (perfect discrimination between targets and non-targets). Composite A′ scores were calculated using A′ and median reaction time (RT) in response to target stimuli. Composite A′ was used to characterize behavior. As a principled combination of both aspects of task performance (discrimination and RT), the use of composite A′ accounts for speed/accuracy trade-offs in processing and diminishes the influence of strategy effects (McNamara and Scott, 2001). The percent difference between mean pre-intervention and post-intervention performance across n-back tasks and within each n-back task (training gain) was calculated for all subjects. These behavioral measures were calculated to serve as a parsimonious account of within-subjects behavioral changes after CCT on an untrained task, enabling the investigation of transfer effects.

#### ERP Analyses

**FIGURE 4:**
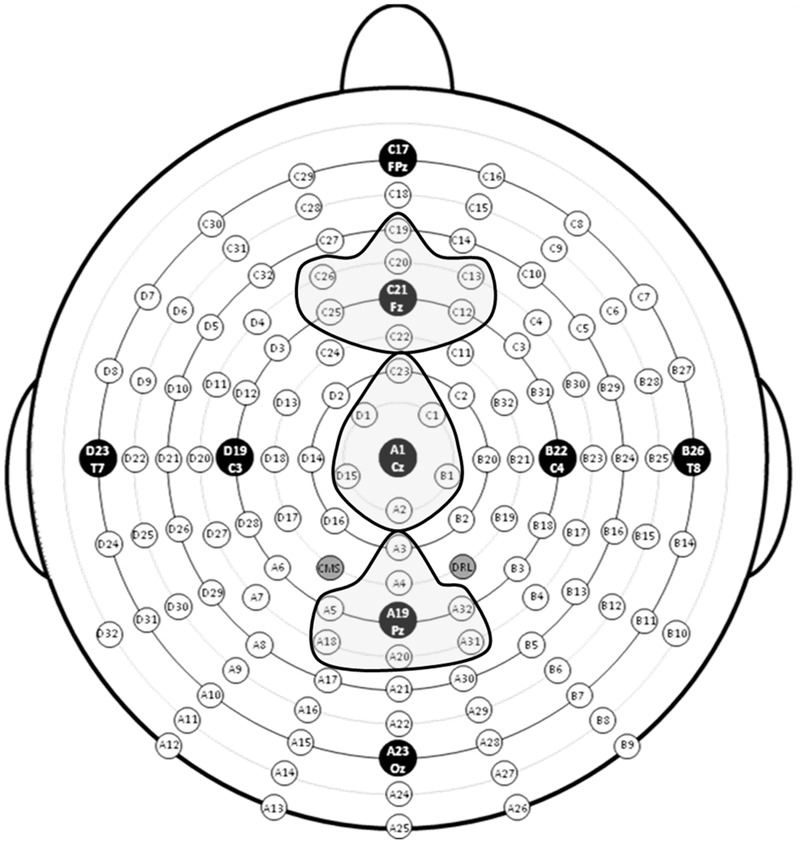
**Electrode montage with regions of interest (ROIs) highlighted**.

EEG data were analyzed using ERPLAB (Lopez-Calderon and Luck, 2014) and EEGLAB (Delorme and Makeig, 2004) toolboxes that operate within the MATLAB framework. Raw EEG data were resampled to 256 Hz and referenced off-line to the algebraic average of the right and left mastoids. EEG signals were filtered using an IIR bandpass filter with a bandwidth of 0.03-30 Hz (12 dB/octave roll-off for all). Individual channels that revealed, upon visual inspection, a consistently different pattern of activity from surrounding channels were corrected with the EEGLAB interpolation function. Eye artifacts were removed through independent component analysis. EEG epochs of target stimuli followed by correct responses (target hits) during the 0-back, 1-back, and 2-back tasks were averaged separately. The sampling epoch for each trial lasted for 1200 ms, including a 200 ms pre-stimulus period that was used to baseline correct the ERP epochs. Trials were discarded from the analyses if they contained baseline drift or movement artifacts greater than 90 μV. Only trials with correct responses were analyzed. Subjects were excluded from further analyses if more than 20% of epochs were automatically rejected using a ±90 μV artifact rejection threshold. Three regions of interest (ROIs), anterior, central, and posterior, were created by averaging clusters of channels centered at midline electrode sites Fz, Cz, and Pz (see **Figure 4**). To index the overall amplitude and scalp distribution of the P3 response to target n-back events, activity was measured at the three ROIs. Responses at the Fz cluster were interpreted as reflecting a greater contribution of P3a activity and at the Pz cluster a greater contribution of P3b activity (Polich, 2007; Alperin et al., 2014).

## Results

### Participants

Forty-one subjects (mean age 75.8) enrolled in the adaptive and control CCT interventions of the experiment; 35 subjects (mean age 75.7) are included in the current study. One subject assigned to control CCT was excluded due to clinical depression. Two subjects assigned to adaptive CCT dropped out of the study, one before completing the first visit and the other after 10 training sessions. Three additional (adaptive CCT: *n* = 1, control CCT: *n* = 2) subjects completed all lab visits and the entire training period, but were excluded from the current study due to inadequate ERP data (too few trials for ERP measurements) (see **Figure 2**). The mean age (77.3) and years of education (16.4) of excluded and withdrawn subjects are both within 1 SD of included subjects. *T*-tests and *X*^2^ tests were used to investigate differences between CCT groups in terms of their demographic characteristics and baseline neuropsychological test scores. There were no differences between CCT groups on age, sex, MMSE scores, days elapsed between training and post-CCT testing, or any of the baseline neuropsychological tests. There were differences between CCT groups on AMNART IQ and years of education (see **Table 1**).

**Table 1:** Pre-CCT demographic variables and neuropsychological percentile scores.

Pre-CCT session	Adaptive (*mean* (*SD*))	Control (*mean* (*SD*))	*p*
Sex (female:male)	12:5	15:3	0.37
Age (years)	74.47 (6.26)	76.84 (5.95)	0.24
Years of education	18.65 (2.98)	16.78 (2.05)	**0.04**
Training/post-CCT days elapsed	4.06 (2.73)	3.67 (1.37)	0.60
MMSE	29.41 (0.71)	28.89 (1.68)	0.24
AMNART IQ	123.59 (4.00)	119.33 (5.86)	**0.02**
WMS-III logical memory II recall	86.26 (23.41)	84.89 (16.76)	0.84
Boston naming test	72.65 (16.67)	65.56 (22.86)	0.30
Trails A	59.76 (28.04)	51.30 (27.00)	0.37
Trails B	56.12 (34.02)	62.67 (24.33)	0.52
WAIS-IV digit symbol	75.56 (23.55)	72.83 (24.33)	0.74
COWAT fluid	71.82 (26.42)	72.20 (26.56)	0.97
COWAT categorical	56.59 (27.87)	57.22 (28.61)	0.95
Mean percentile score	68.40 (16.13)	66.67 (17.45)	0.76

*Significant *p* values are bold.*

### Behavior

A 2 session (pre-CCT, post-CCT) × 3 task (0-back, 1-back, and 2-back) × 2 CCT group (adaptive CCT, active control CCT) ANOVA was performed on the A′ composite measure. An effect of n-back task, *F*(2,66) = 132.20, *p* < 0.001, η^2^ = 0.800, revealed a decrease in performance as task load increased (0-back > 1-back > 2-back). There were no differences between CCT groups, *F*(1,33) = 2.02, *p* = 0.165, and no interaction between session and CCT group, *F*(1,33) = 0.23, *p* = 0.638.

### Event-Related Potentials

#### Local Peak Latency

**FIGURE 5:**
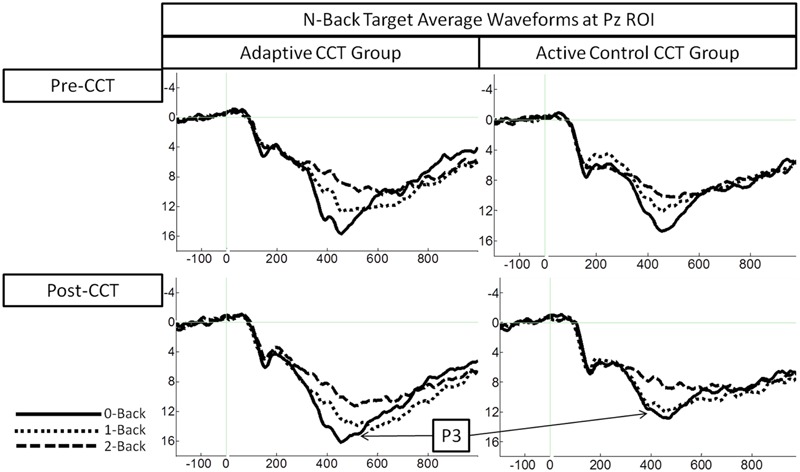
**Average waveforms plotted at Pz ROI**.

**FIGURE 6:**
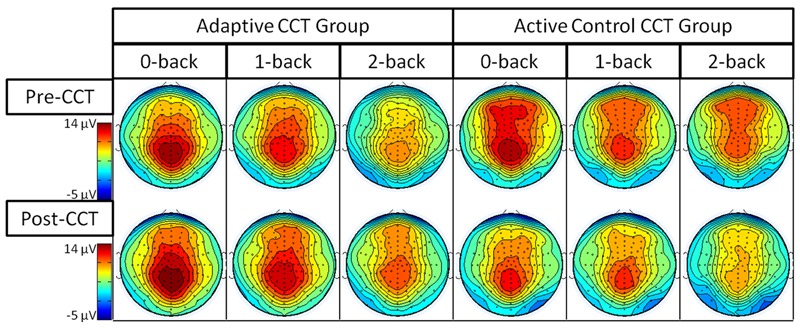
**Topographic scalp plots of P3 mean amplitude**.

Event-related potentials in response to target hits during 0-back, 1-back, and 2-back tasks were analyzed. Peak latency of the P3 was measured at Fz ROI, Cz ROI, and Pz ROI over a 250 ms window (350-600 ms) selected based on visual inspection of the grand average waveform collapsed across group to reduce bias. Local peak latency measurements were analyzed via a 2 session × 3 task × 3 ROI × 2 CCT group ANOVA, which revealed a main effect of n-back task, *F*(2,66) = 8.87, *p* < 0.001, η^2^ = 0.21, such that P3 latency increased as task difficulty increased. P3 latencies during the 0-back and 1-back did not differ significantly, (*p* = 0.076) though P3 latency during the 1-back tended to be longer than during the 0-back. P3 latency during the 2-back was longer than both 0-back and 1-back (*p*s < 0.02). Notably, there was no effect of session, *F*(1,33) = 0.31, *p* = 0.582, or session × CCT interaction, *F*(1,33) = 0.653, *p* = 0.425. See **Figure 5** for an illustration of average waveforms at the Pz ROI and **Figure 6** for topographic scalp plots of the target P3.

#### Mean Amplitude

Amplitude measurement windows for each task were defined as the 150 ms window centered on the mean P3 local peak latency during each n-back task (0-back *M* = 475 ms, 1-back *M* = 491 ms, and 2-back *M* = 512 ms). A 2 session (pre-CCT, post-CCT) × 3 n-back (0-back, 1-back, and 2-back) × 3 ROI (Fz, Cz, Pz) × 2 CCT group (adaptive CCT, active control CCT) ANOVA was performed on mean amplitude measurements. See **Table 2** for all main effects and interactions. There was no main effect of session or of CCT group. However, there was an interaction between session and CCT group, which was driven by different effects of session within each CCT group. In the adaptive CCT group, the effect of session, *F*(1,16) = 12.58, *p* = 0.003, η^2^ = 0.48, revealed an increase in target P3 amplitude between the pre-CCT and post-CCT sessions. In the active control CCT group, the effect of session, *F*(1,17) = 5.25, *p* = 0.035, η^2^ = 0.24, revealed a post-CCT decrease in target P3 amplitude. P3 amplitude did not differ between CCT groups during either session (*p*s > 0.2).

**Table 2:** Average waveforms - 2 Session × 3 N-Back × 5 Electrode Site × 2 CCT Group ANOVA - Main Effects and Interactions.

ANOVA main effects/interactions	*df*	*F*	*p*	Bonferroni-Holm *p*	$\eta_{p}^{2}$
N-Back	2,66	25.18	0.000	**0.000**	0.43
N-Back × ROI	4,132	15.73	0.000	**0.000**	0.32
Session × CCT group	1,33	13.06	0.001	**0.013**	0.28
ROI	2,66	7.626	0.002	**0.018**	0.19
Session × ROI × CCT group	2,66	3.951	0.038	0.423	0.11
Session × ROI	2,66	2.542	0.105	1.000	0.07
Session × N-back × CCT group	2,66	1.294	0.279	1.000	0.04
ROI × CCT group	2,66	1.092	0.338	1.000	0.03
N-Back × ROI × CCT group	4,132	1.041	0.376	1.000	0.03
Session × N-back × ROI	4,132	0.829	0.437	1.000	0.02
N-Back × CCT group	2,66	0.792	0.445	1.000	0.02
CCT group	1,33	0.169	0.684	1.000	0.01
Session	1,33	0.142	0.709	1.000	0.00
Session × N-back × ROI × CCT group	4,132	0.292	0.740	1.000	0.01
Session × N-back	2,66	0.191	0.798	0.798	0.01

*Significant *p* values are bold.*

**FIGURE 7:**
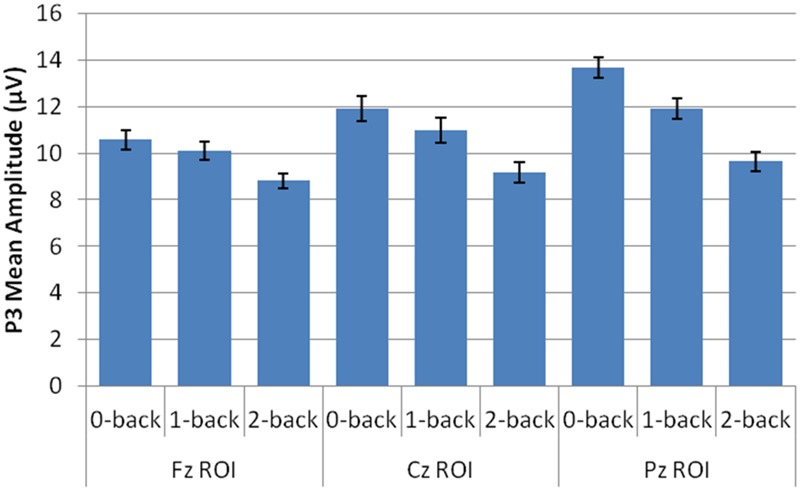
**Amplitude at three ROI during three tasks**.

The interaction between session and CCT group was modified by ROI. While the effect of session did not vary across ROI for the adaptive CCT group, the effect of session for the control CCT group was only observed at anterior locations (Fz ROI, *F*(1,17) = 7.89, *p* = 0.012, η^2^ = 0.32; Cz ROI, *F*(1,17) = 4.23, *p* = 0.055; Pz ROI, *F*(1,17) = 2.32, *p* = 0.146. The effect of ROI revealed that target P3 amplitude was greatest at Pz ROI (*p*s < 0.03) but not reliably different between Fz ROI and Cz ROI (*p* = 0.072). The main effect of n-back task was due to a decrease in P3 amplitude as WM load increased; P3 amplitude during 0-back was greater than during 1-back, which, in turn, was greater than during 2-back (*p*s < 0.01). The main effect of ROI was modified by n-back. As n-back task load increased, the P3 response became more anteriorly distributed [0-back: Fz ROI < Cz ROI < Pz ROI, *p*s < 0.02; 1-back: Fz ROI < Pz ROI (*p* = 0.007), Fz ROI = Cz ROI (*p* = 0.096), Cz ROI = Pz ROI (*p* = 0.062); 2-back: Fz ROI = Cz ROI = Pz ROI (*p*s > 0.1)] (**Figure 7**). Non-significant interactions were also informative; the interaction between session and CCT group was not modified by n-back load. This result indicates that the magnitude of within-group session differences did not vary across n-back task WM load.

Previous work with older adults (Daffner et al., 2011a) revealed that, in contrast to subjects who performed in the bottom half on the n-back task, those who performed in the top half demonstrated an increase in P3 amplitude as WM load increased. To determine if high and low performing subjects in the current study differed in their pattern of P3 response as load increased, a median split of the group was carried out based on mean A′ composite score collapsed across pre-intervention and post-intervention sessions. Subjects who performed above the median were classified as high performers and those who performed below the median as low performers. An ANOVA on P3 amplitude measurements with an additional group variable defined by n-back task performance (2 session × 3 n-back task × 3 ROI × 2 CCT Group × 2 n-back performance group (high /low)) was conducted. An effect of n-back task, *F*(2,62) = 24.91, *p* < 0.001, η^2^ = 0.446, indicated that P3 amplitude decreased as n-back task load increased. Of particular relevance to this analysis, there was no interaction between performance group and n-back task, *F*(2,62), *F* = 1.23, *p* = 0.295, indicating that both high and low performing subjects demonstrated a load-related decrease in target P3 amplitude during n-back tasks.

### Behavioral/Electrophysiological Correlations

**FIGURE 8:**
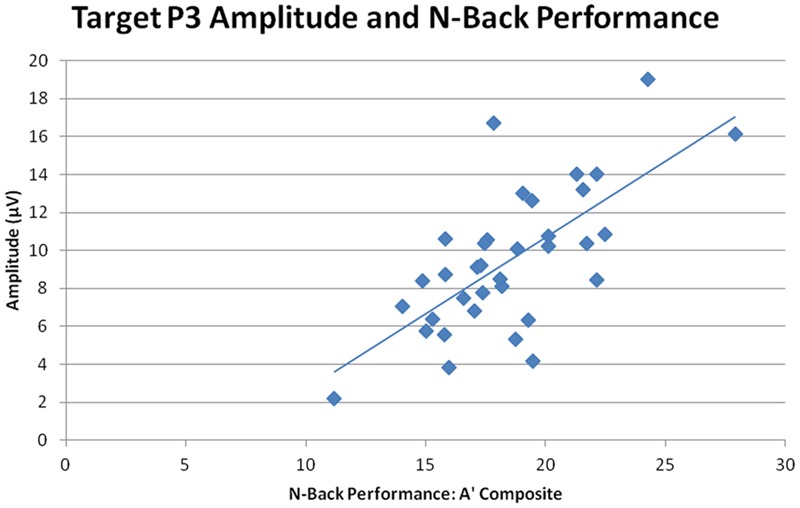
**N-Back performance and target P3 amplitude**.

Overall target P3 amplitude (collapsed across sessions, n-back tasks, and ROIs) directly correlated with n-back performance, *r* = 0.770, *n* = 35, *p* < 0.001, indicating that larger target P3 amplitude was correlated with better performance across all three n-back tasks (smaller RTs and higher A′ scores, see **Figure 8**). Post-CCT change in target P3 was calculated as the percent difference in target P3 amplitude between pre-CCT and post-CCT sessions. Post-CCT change in P3 amplitude correlated with training gain during 0-back, *r* = 0.369, *n* = 35, *p* = 0.029, 2-back, *r* = 0.635, *n* = 35, *p* < 0.001, and collapsed across tasks, *r* = 0.357, *n* = 35, *p* = 0.035. While post-CCT change in amplitude and training gain did not correlate when limited to the 1-back task, *r* = 0.311, *n* = 35, *p* = 0.069, there was a strong tendency toward a relationship between performance improvement and P3 amplitude increase. Taken together, these results indicate that larger post-CCT target P3 amplitude increases were associated with larger post-CCT gains in n-back task performance, and that this relationship was strongest at the highest level of task load.

A similar set of correlations was investigated for P3 latency measurements. Overall target P3 latency (collapsed across sessions, n-back tasks, and ROIs) did not correlate with n-back performance, *r* = -0.210, *n* = 35, *p* = 0.225. Post-CCT change in target P3 latency was calculated in a manner similar to post-CCT change in target P3 amplitude. Post-CCT change in P3 latency did not correlate with training gain during any n-back task or across tasks, |r| ’s < 0.3, *n* = 35, *p*s > 0.1.

While there were CCT group differences on AMNART IQ scores and years of education, no group differences were found in electrophysiological or behavioral measures during either session. To further investigate any influence of these pre-CCT demographic group differences, we examined whether there were correlations between electrophysiological and performance measures, collapsed across all n-back levels. Neither years of education nor IQ correlated with training gain or target P3 amplitude averaged across tasks, sessions, and ROIs (overall target P3) (*r*s < 0.3, *p*s > 0.1). Across all n-back tasks and both sessions, n-back performance did not correlate with IQ, *r* = 0.135, *n* = 35, *p* = 0.441, but was directly correlated with years of education, *r* = 0.378, *n* = 35, *p* = 0.025.

## Discussion

The major findings of the study can be summarized thusly: (1) In contrast to the control CCT, adaptive CCT was associated with an augmentation of the P3 amplitude to target events in the experimental n-back task. The enhanced P3 was observed at both anterior and posterior sites, consistent with augmentation of both P3a and P3b activity. (2) The increase in the size of the P3 component in response to adaptive cognitive training was of similar magnitude across all n-back task levels. (3) The size of the P3 component strongly predicted performance on the n–back tasks; the larger the P3 amplitude, the better the performance. (4) Training–related increases in P3 amplitude strongly predicted improvement in performance on the n-back tasks, especially under the hardest condition; the greater the increase in P3 amplitude between sessions, the greater the improvement in performance on n-back tasks. (5) There was no difference in task performance between the two CCT groups, and neither group showed transfer effects of CCT on untrained task performance. (6) There was no difference between sessions in P3 latency for either CCT group.

Our finding of a training-related augmentation in neural activity, as indexed by P3 amplitude, differs from other reports in the literature, which have tended to show a reduction in neural activity after cognitive training (Belleville et al., 2014; Heinzel et al., 2014; Vermeij et al., 2016). One potential contributing factor to this discrepancy may be that the current investigation studied an older sample of subjects than in many previous investigations (Brehmer et al., 2011; Belleville et al., 2014; Vermeij et al., 2016). For example, our subjects were, on average, older than subjects in the Brehmer et al. (2011) study by more than 11 years, and older than the subjects in the Belleville et al. (2014) or Vermeij et al. (2016) studies by more than 6 years. In addition, this group of subjects was very well educated and scored at high levels on neuropsychological tests – the average percentile score based on age-appropriate norms for all NP tests indicated that subjects performed in the top third of individuals in their age group.

We predicted that adaptive CCT would be associated with improved performance on the untrained transfer task, and also that neural activation, as indexed by P3 amplitude on the experimental task, would increase at high load and decrease at low load (Brehmer et al., 2012; Shinaver et al., 2014). This pattern of response can be conceptualized using the CRUNCH hypothesis of cognitive aging (Reuter-Lorenz and Cappell, 2008; Schneider-Garces et al., 2010). If adaptive CCT increased processing capacity, the inverted U shaped curve relating neural activity and load would be shifted to the right, allowing individuals to effectively perform low load tasks by allocating fewer resources, while raising the WM load threshold when resources become exhausted and neural activation begins to decrease (Daffner et al., 2011a) (**Figure 1C**). In contrast to our prediction, no post-CCT performance changes were found for the adaptive CCT group, and we did not observe the predicted decrease in activation at low levels of WM load paired with an increase at higher levels of load. Rather, we found an increase in P3a and P3b activity after adaptive CCT across all 3 task loads (**Figure 1B**). After active control CCT, we expected no change in either untrained task performance or electrophysiological measures. There was no difference in performance between pre-CCT and post-CCT sessions, a post-CCT decrease in activation of WM process indexed by P3 amplitude was found. This post-CCT decrease in P3 amplitude was limited to anterior regions, indicating that the P3a component may have decreased while the P3b did not vary across sessions for the control CCT group. Taken together, our results suggest that in response to CCT without adaptively changing difficulty (i.e., the control condition), there is a tendency to decrease the activation of executive control/attentional processes, but there is no impact on WM/updating processes. This pattern of response was unlike the response to adaptive CCT that promoted the allocation of additional resources for both P3a and P3b processes, which was shown to be associated with better task performance.

**FIGURE 9:**
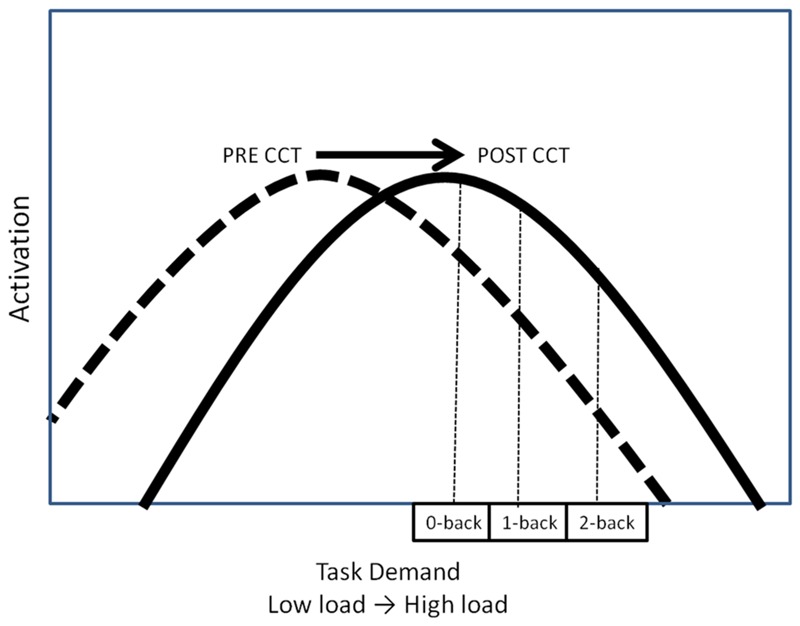
**Hypothetical task-demand/resource utilization curve**.

Both low-performing and high-performing subjects in the current study exhibited a decrease in P3 amplitude as the n-back task demands were augmented. This pattern contrasts with what was observed in a previous study of older adults (Daffner et al., 2011a), who were younger (mean age = 72.1) than the subjects in the current experiment. Using the same experimental task (Daffner et al., 2011a), we found that the high performing older subjects demonstrated an increase in P3 amplitude as n-back WM load increased. The results of the current study suggest that at the lowest task load (0-back), subjects had already exceeded their maximal allocation of resources (‘crunch’ point) for attention and updating processes, possibly due to their generally older age (Daffner et al., 2011b). If this explanation is correct, then the post-CCT generalized increase in activation in the current study may also be characterized as a rightward shift of the task demand/resource utilization curve, as predicted (**Figure 9**).

Adaptive CCT not only involves a variety of tasks that require different WM processes (spatial and/or verbal tasks, maintenance, manipulation), but also augments WM load over time. This approach should promote the adoption of new strategies (Olesen et al., 2004; Klingberg, 2010) rather than the reliance on automatic processes that may have been adequate to manage lower levels of demand. Subjects were given no explicit instructions about carrying out the CCT tasks. Both the independent generation of new task strategies and the frequent switching between tasks require executive control. Training these operations may have led to increased post-CCT activation of networks involved in controlled processing during the untrained task, as indexed by P3 amplitude. The static nature of WM demands in the active control CCT is unlikely to have led to this same demand for executive control, perhaps resulting in unchanged or slightly decreased activation during the second session (Brehmer et al., 2011; Belleville et al., 2014).

Previous studies have interpreted reduced neural activation to represent an improvement in processing efficiency, and therefore as an advantageous adaptive change (Brehmer et al., 2011; Belleville et al., 2014). The current study does not lead to the same conclusion. Target P3 amplitude strongly correlated with performance, such that individuals who generated a larger amplitude P3 response to targets performed the n-back tasks both more quickly and accurately. Of note, the behavioral variable used to measure performance (A′ composite) utilizes both RT and A′ (a measure of accuracy), thereby accounting for accuracy / RT tradeoffs (McNamara and Scott, 2001).

To investigate how performance changes on the transfer task were related to electrophysiological changes after CCT, without regard to whether training reflected adaptive or control conditions, we examined the relationship between post-CCT training gain, a measure of change in n-back performance, and post-CCT change in amplitude of electrophysiological measures. Performance improvement on the transfer task, as measured by training gain, was positively correlated with the post-CCT change of target P3 amplitude. This allows us to conclude that P3 amplitude in response to targets, which increased after adaptive CCT, is not only directly correlated with performance on transfer tasks, but also that larger training-related increases of target P3 amplitude are associated with larger gains in task performance. For example, on the 2-back task, the training-related increase in P3 amplitude accounted for over 40% of the variance in behavioral training gain. Our findings suggest that future interventions aimed at developing ways to augment resources appropriated for operations indexed by the P3a and P3b may be particularly successful at promoting transfer effects.

P3 latency has been characterized as a measure of speed of information processing (Kutas et al., 1977; Polich, 1996). The load related increase in P3 latency was expected, given reports in the P3 literature (Polich and Kok, 1995; Verleger, 1997) as well as the results of our own work (Daffner et al., 2011a). The absence of session effects or correlations between post-CCT P3 latency and performance changes strengthens the argument that improvements in task performance were more closely linked to greater engagement and resource allocation for WM updating (as indexed by P3 amplitude) than to speed of stimulus processing.

The observed influence of CCT in the current study provides mixed support for previous findings in the literature. Future investigations may be informed by some of the unanticipated results of the current study, especially the lack of behavioral transfer effects in the presence of post-CCT changes in neural activation. The absence of a clear link between performance improvements and changes in neural activity during easier tasks (e.g., CCT group × session interactions for both electrophysiological and behavioral measures) may be driven by a ceiling effect. While activation increased after training, high performance on low load tasks during the pre-CCT session may have limited the possibility of performance improvements on the untrained task. Additionally, the current study includes considerably older subjects than other similar studies. Younger- and older-old groups may respond to training differently. Whereas young-old adults may be able to increase automaticity and reduce cortical activation after training while maintaining performance (Belleville et al., 2014), older-old individuals may continue to rely on executive control operations even in response to low load conditions, and utilize training to expand their capacity to recruit additional cortical resources to manage task demands (De Sanctis et al., 2009; Daffner et al., 2011b). To test this hypothesis, future studies need to include a wider age range of older subjects.

Given the relatively low cost and feasibility of in-home, computer-mediated cognitive exercises, there is also growing interest in evaluating the impact of this kind of training on patents with neurologic and non-neurologic conditions (Barnes et al., 2009; Fisher et al., 2009; Simon et al., 2012; Von Ah et al., 2012; Edwards et al., 2013; Huckans et al., 2013; Vermeij et al., 2016). Including ERPs in such investigations, as was done in the current study, can help to determine underlying mechanisms of change and perhaps identify which patients might benefit most from such efforts.

In summary, adaptive CCT in older adults was associated with increased neural activity, as measured by the P3 component. Greater neural activity underlying attention and categorization/updating processes was associated with better overall performance on WM tasks, and training-related increases in P3 activity strongly predicted behavioral improvement, especially under the most demanding conditions. It remains to be determined why no direct link between WM training and improved task performance was observed.

## Author Contributions

KD, ER, PH, AM designed the study. ET, BA, ER, collected the data. ET analyzed data, drafted the initial manuscript, and created figures. ET, BA, PH, AM, and KD edited the manuscript.

## Conflict of Interest Statement

The authors declare that the research was conducted in the absence of any commercial or financial relationships that could be construed as a potential conflict of interest.
